# A size-dependent structural evolution of ZnS nanoparticles

**DOI:** 10.1038/srep14267

**Published:** 2015-09-18

**Authors:** Mohammad Khalkhali, Qingxia Liu, Hongbo Zeng, Hao Zhang

**Affiliations:** 1 Department of Chemical and Materials Engineering, University of Alberta, Edmonton, Alberta T6G 2V4, Canada

## Abstract

Recently, ZnS quantum dots have attracted a lot of attention since they can be a suitable alternative for cadmium-based quantum dots, which are known to be highly carcinogenic for living systems. However, the structural stability of nanocrystalline ZnS seems to be a challenging issue since ZnS nanoparticles have the potential to undergo uncontrolled structural change at room temperature. Using the molecular dynamics technique, we have studied the structural evolution of 1 to 5 nm freestanding ZnS nanoparticles with zinc-blende and wurtzite crystal structures. Simulation results revealed that relaxed configurations of ZnS nanoparticles larger than 3 nm consist of three regions: a) a crystalline core, b) a distorted network of 4-coordinated atoms environing the crystalline core, and c) a surface structure made entirely of 3-coordinated atoms. Decreasing the size of ZnS nanoparticle to 2 nm will cause the crystalline core to disappear. Further reducing the size will cause all of the atoms to become 3-coordinated. Dipole moments of zinc-blende and wurtzite nanoparticles are in the same range when the nanoparticles are smaller than 3 nm. Increasing the size makes dipole moments converge to the bulk values. This makes zinc-blende and wurtzite nanoparticles less and more polar, respectively.

II-VI semiconductor nanomaterials are of great technological interest due to their unique optoelectronic properties. The quantum confinement effect makes it possible to tune the frequency range of emitted light (colour) from semiconductor nanostructures, usually known as quantum dots (QDs), by controlling their sizes^1^. Because they also have wide absorption and luminescent efficiency, a high resistance to photobleaching, and high chemical stability, quantum dots are an ideal candidate for use in biomedical imaging applications, where they can be used in place of organic fluoroscopes^2^. Owing to the rapid growth of nanotechnology, a variety of synthesis procedures have been proposed to fabricate II-VI semiconductor nanoparticles (NPs) in different sizes, shapes and structures^3^. The typical QD size is smaller than 5 nm mainly to make the best use of the quantum confinement effect. Moreover, studies have shown that the level of toxicity of QDs also decreases with decreasing sizes. It has been reported that large QDs are generally accumulated for several months in the reticuloendothelial system, such as liver, spleen and lymphatic system, but QDs smaller than 5 nm could be removed quickly by the kidney^4^.

The rapid development of QD technology has raised serious concerns about its applicability, mainly because the most studied II-VI semiconductor nanomaterials contain cadmium (CdSe, CdTe and CdS), which is known to be highly carcinogenic for living systems^5^. Various modifications such as adding a ZnS shell or polyethylene glycol (PEG) coating have been suggested to modify QDs with toxic elements. However, the cytotoxicity of Cd containing QDs is still a major concern^6^. Zn-based QDs such as ZnS have been introduced as a suitable alternative for QDs with Cd components since Zn is considered to be an essential biological element^7^. Furthermore, the band gap of ZnS is more than 1 eV wider than the others in the II-VI family, which enables a wider spectrum to be tuned by varying the size of ZnS nanostructures. However, the structural stability of nanocrystalline ZnS has been shown to be a challenging issue limiting its applicability since a ZnS NP has the potential to undergo uncontrolled structural changes^8^.

At standard temperature and pressure (STP: 298 K and 1 bar), bulk ZnS exists in two crystal structures: zinc-blende (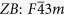) and wurtzite (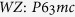). ZB structure has slightly lower energy at standard condition while WZ is more stable above 1020 °C. At higher pressures (above 15 GPa), the high density rocksalt structure (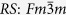) becomes more stable. It has been shown that at the nanoscale, the phase transformation behaviour of ZnS can deviate greatly from bulk. For ZnS nanostructure, the temperatures as low as 400 °C^9^ and 250 °C^10^ have been reported for the ZB-to-WZ phase transformation at standard pressure and 1 GPa, respectively. However, pressure-induced transformations to the RS phase have been shown to increase to 19.6 GPa in ZnS NPs^11^.

The structural transformation of ZnS NPs has also been reported at room temperature. Zhang *et al.* showed that reversible structural transformations at room temperature could be induced by the absorption-desorption of methanol and water^12^. They found that absorption of water to the surface of ZB ZnS NPs increased crystallinity, a finding also supported by molecular dynamics (MD) simulation results^13^. MD has also been used to study the structural relaxation of a freestanding 3 nm ZnS NP at 300 K^14^ as well as the aggregation behaviour of NPs with the same size^15^. A phase transformation from ZB to WZ structure was reported in both cases. Haung and Banfield have shown that WZ grows on the surface of coarsened ZB particles during aggregation at 500 K^16^. The crystal growth of WZ, however, was kinetically controlled by WZ-ZB interface radius, and no pure WZ particles were observed in coarsened samples.

Using MD and DFT calculations, Hamad and Catlow studied (ZnS)_n_ clusters with sizes ranging from 1 to 4 nm (18 < *n* < 512)^17^. They showed that small clusters (*n* < 80) adopted bubble-like or onion-like structures, which predominantly consisted of arrangements of 3-coordinated atoms. Crystal structures of large clusters (*n* = 256 *and n* = 512) obtained by simulated annealing, mainly consisted of 4-coordinated atoms but deviated from the two bulk phases of ZnS found in nature and were shown to be similar to the BCT zeolite structure^17^. Applying surface energies calculated using first principle computer simulations into a thermodynamic model, Barnard *et al.* studied the effect of shape and size on the stability of ZnS ZB^18^ and WZ^19^ NPs. They found that the rhombic dodecahedron shape, enclosed entirely by non-polar {110} facets, was the most stable ZnS shape regardless of the size of the particle^18^. However, it has been shown that adding polar facets to ZB nanostructures would make core-shell crystalline/amorphous structure thermodynamically favourable. Deviation from the rhombic dodecahedron shape enabled some thermodynamic paths from ZB to WZ transformation by decreasing the size^19^. In contrast, high energy WZ nanostructures with low prism aspect ratios or lower indexed pyramidal capping facets were prone to transform to ZB, especially when the size increased^19^.

In this paper we aim to provide details about how the freestanding ZnS NP size affects structural evolution in standard condition. Structural and configurational evolutions of ZnS NPs were studied using classical MD method. Different structural analyses including radial distribution function (RDF), angular distribution (AD), Honeycutt-Andersen indices, root mean square displacement (RMSD) and coordination number (CN) calculations were performed to characterize the relaxed structures of NPs. In perfect ZB and WZ lattices, each ion is connected to four dissimilar ions with a tetrahedral bond geometry in which all bond angles are equal to 109.47°. Based on bond angles and CN population analyses, we show that the structure of relaxed ZnS NPs bigger than 2 nm consist of three regions: a) a 4-coordinated crystalline core at which Zn and S ions keep their initial tetrahedral arrangement, b) a distorted network of 4-coordinated ions which environs the crystalline core, and c) the surface structure which consists of a network of 3-coordinated ions. The stability and size of each region is highly dependent on the crystal structure and size of the ZnS NP. The 4-coordinated tetrahedral bond structure completely disappears in 2 nm ZnS NPs and decreasing the size down to 1 nm results in a bubble-like structure in which all atoms are 3-coordinated. The effect of this structural evolution on NPs’ dipole momenta (DM) has also been studied since dipole-dipole interactions are of great importance in non-metallic nanoparticles, as they are found to govern the inter-particle interactions which can vary the behaviour of the mixture of NPs from agglomeration to self-assembly into ordered structures. Results of DM calculations show that in relatively smaller NPs where the surface effect is dominant, DM is controlled by the surface structure. Due to the similarity of the surface structure of ZB and WZ NPs, their DM is similar when the size is smaller than 3 nm. However, in NPs bigger than 3 nm, the direction and magnitude of DM approach the bulk values. This implies that ZB NPs become less polar while the polarity of WZ NPs increases due to the considerable polar nature of WZ lattice.

## Results

**Figure 1:**
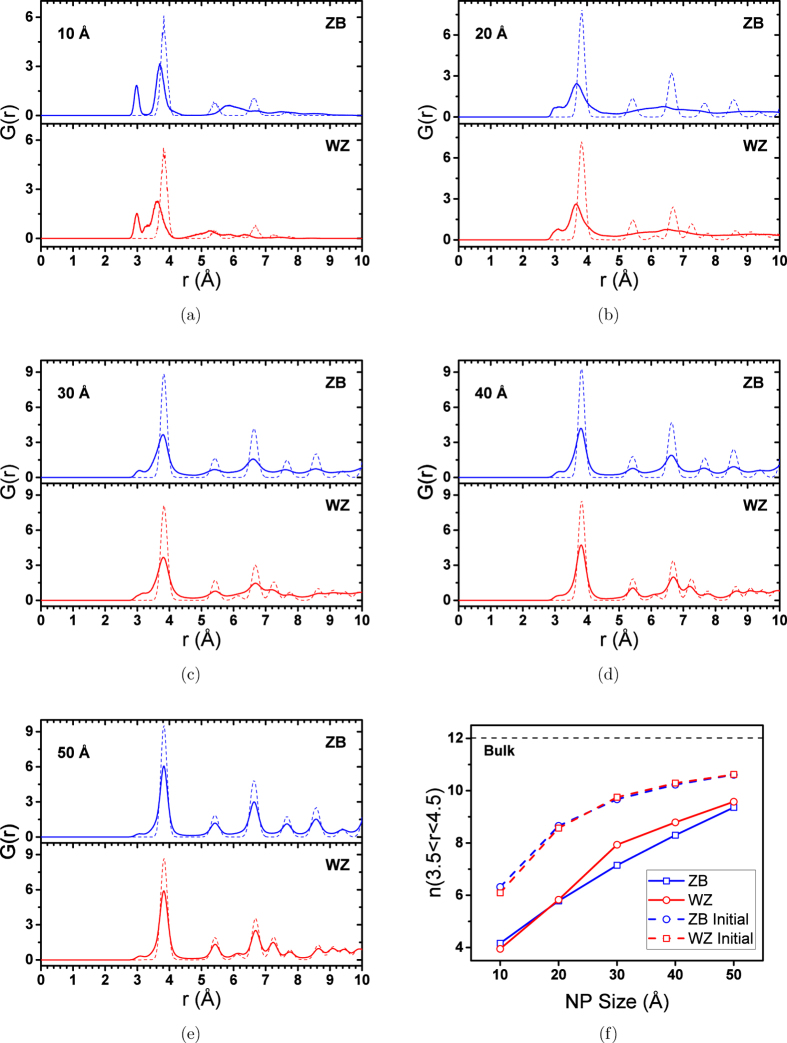
RDF plots of Zn-Zn pairs for (a) 10, (b) 20, (c) 30, (d) 40 and (e) 50 NPs. (**f**) shows the number of Zn-Zn pairs whose distances are between 3.5 and 4.5 Å, 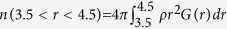. Dashed lines show the corresponding plots for the NPs with the same size but with an unrelaxed structure.

**Structural Evolution.**    *The Radial Distribution Function.*    The radial distribution function of atom B in the distance *r* from atom A (*g*(*r*)) can be readily calculated from MD trajectories as,


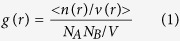


where *n*(*r*) is the number of B atoms found in the shell between *r* and *r* + *dr*, *v*(*r*) is the volume of the shell, *N*_*A*_ and *N*_*B*_ are the total number of atom A and atom B in the system, *V* is the total volume of the system, and 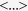 denotes the ensemble average. For the small *dr*, *v*(*r*) can be approximated as *v*(*r*) = 4*πr*^2^*dr* and equation (1) can be rewritten as


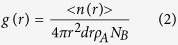


where *ρ*_*A*_ is the number density of atom A in the system. In a special case where A and B are the same, *N*_*B*_ is equal to *N* − 1, where *N* is the total number of A atoms in the system. Some care should be taken into consideration when calculating RDF for a freestanding NP. As the NP has undercoordinated atoms on its surface, its *n*(*r*) is lower than what is calculated for the bulk. This means the intensity of RDF peaks of NPs are lower than bulk even for the initial structure. It is especially important for 1 and 2 nm NPs because of their high surface-to-volume ratio. While the position of RDF peaks of relaxed NPs can be compared with the bulk, making a direct comparison between the intensities of RDF peaks is not accurate. A RDF diagram of a NP with the same size and at the same temperature but with no structural relaxation may be used as a reference to study the crystal structure change in the relaxed NP. This reference RDF is made for each NP by cutting a sphere with the same size as the NP from the trajectories of 1 ns NPT simulation of the periodic bulk structure at 300 K and 0 atm. One other issue in the RDF calculations of freestanding NPs is that the volume is not defined for non-periodic systems. As a result *ρ* should be defined carefully. In our RDF calculations, we used the number density of the bulk ZnS as a normalizing factor, *ρ*. This puts the intensities of NPs’ RDF peaks on the same order of magnitude with each other as well as the bulk. Figure 1 shows the calculated RDFs for Zn-Zn pairs in ZB and WZ NPs with different sizes. RDF plots of unrelaxed structures are calculated as explained above. *n*(3.5 < *r* < 4.5), the number of Zn-Zn pairs whose distances are between 3.5 and 4.5 (area under the first RDF peak), is also shown in Fig. 1.

A general trend of losing crystallinity with decreasing the size of NPs can be recognized in RDF diagrams in Fig. 1. In ideal ZB and WZ structure, the first peak of Zn-Zn RDF diagram appears at 3.84 Å. For 10 and 20 Å NPs the position of this peak is changed and the Zn-Zn distance is reduced. All other peaks after 3.84 Å seem to fade away in these small NPs. These two features of RDF diagrams of 10 and 20 Å NPs suggest that these NPs lose their inital crystal structures after relaxation. NPs bigger than 20 Å show the crystalline RDF diagrams, suggesting that considerable portion of the NP structure keeps the crystalline order. The difference between RDF peaks intensities of unrelaxed and relaxed NP decreases as the size of the ZnS NP increases. This suggests that increasing the size of the ZnS NP enhances its crystal structures after relaxation. There is a tiny peak that emerges before the first neighbour’s peak and its intensity increases by decreasing the size of the NPs. In other words, the intensity of this peak increases while the crystal structure loses stability. Hamad *et al.* reported the Zn-Zn RDF diagrams similar to what we observed for 10 and 20 Å NPs for small clusters which adopt double-bubble structures^20^. They related the appearance of the first tiny peak to the formation of 4-rings of 3-coordinated atoms in the bubble-like structures.

Figure 1f shows 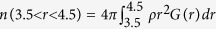 for ZnS NPs with different sizes as well as bulk structures. For a periodic system, this quantity is equal to the coordination number. Due to the undercoordinated surface atoms, this number is lower for NPs and decreases by decreasing the size of the NP (because the surface to volume ratio is higher in smaller NPs). The difference between the *n*(3.5 < *r* < 4.5) of relaxed and unrelaxed configurations is an indication of the deviation of the relaxed structure from the ideal NP structure.

**Figure 2:**
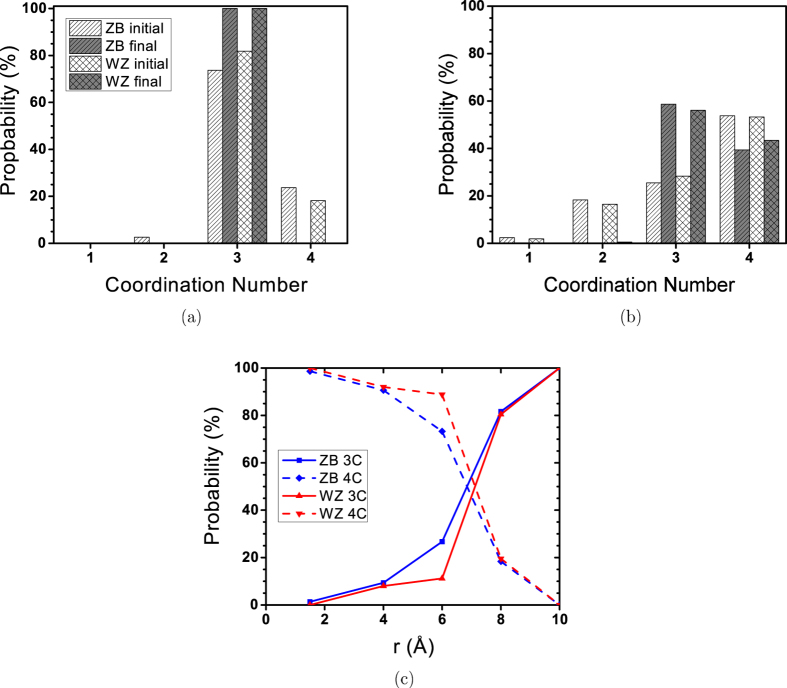
Distribution of atomic coordination number in initial and final configurations of (a) 10 Å and (b) 20 Å NPs. (**c**) Shows the probability of finding 3− and 4-coordinated atoms in 20 Å ZB and WZ NPs as a function of distance from the centre of NP.

*1 and 2 nm NPs.*    As mentioned before, the main characteristic of the bubble and onion like double bubble (where a network of 4-coordinated atoms connects the inner and outer bubble clusters) structures, is that the majority of atoms are three-coordinated. The coordination number (CN) of atom A was calculated by counting the number of dissimilar atoms (B) within a 3 Å distance from atom A. The 3 Å distance was chosen according to the first and second peaks in the Zn-S RDF diagram of periodic bulk ZnS (corresponding to the first and second dissimilar neighbours), which are 2.35 and 4.5 Å, respectively. Figure 2 represents the results of CN calculations for 10 and 20 Å NPs.

Since the initial configurations were made of perfect ZB and WZ structures, all under-coordinated atoms in the initial configurations (*CN* < 4) are located on the surface. Due to the higher surface-to-volume ratio of 10 Å NP, its initial configuration has more undercoordinated atoms compared to the 20 Å NP. Figure 2a shows that all atoms in 10 Å NP become 3-coordinated after relaxation at 300 K. This confirms the stability of the bubble-like structure for 1 nm ZnS NPs which has been also shown by previous studies^17^,^20^. In contrast, a considerable number of 4-coordinated atoms are detected in the relaxed 20 Å NPs, indicating that it is not likely that the atoms rearranged into a bubble-like structure (Fig. 2b). Figure 2b also shows that all the atoms become 3- or 4-coordinated after the relaxation and there is no 1− or 2-coordinated atoms in the final configuration of 20 Å NPs. The probability of finding 3− and 4-coordinated atoms in 20 Å NPs as a function of distance from the centre of the NP is represented in Fig. 2c. This figure also shows that the majority of atoms located in the cores of NPs (*r* < 5) keep their 4-coordinated structure. For both ZB and WZ NPs, the probability of finding 3-coordinated atoms increases as we approach the surface, but they increase with different rates. It seems the 4-coordinated structure is less stable in the 20 Å ZB NP.

**Figure 3:**
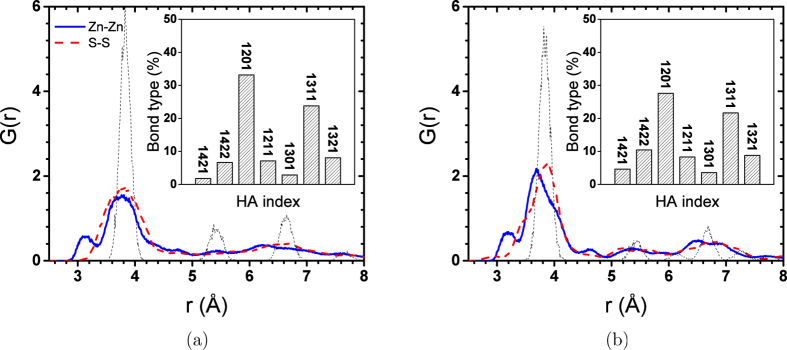
RDF of Zn-Zn and S-S pairs at the core (*r* < 5 Å) of 20 Å ZnS NPs with initial (a) ZB and (b) WZ structures. Inset plots are HA indices for the same NP. Dashed lines show the corresponding plots for the NPs with the same size but with an unrelaxed structure. All calculation were done using trajectories of the last 1 ns of the simulations.

To understand the nature of the 4-coordinated structure at the core of 20 Å NPs, we calculated the RDF for Zn-Zn and S-S pairs as well as the Honeycutt-Andersen (HA) indices^21^ for atoms located in the core of the NPs. Four HA indices are defined as follows: the first index represents the RDF peak number to which the atomic pair of interest belongs and is usually equal to one (first neighbours). The second index is the number of common nearest neighbours of the atomic pair of interest. The third index counts the number of common neighbours which form a bond and the fourth one is used to differentiate clusters with identical sets of the first three indices but different configurations. HA indices can be used to identify the local FCC and HCP arrangements. It has been shown that the 1421 bond type is characteristic of the FCC crystal, while in the HCP structure, 1422 bonds are also predominant^22^. HA indices of Zn-Zn and S-S pairs can be used to track structural changes, since Zn and S atoms have FCC and HCP arrangements in ZB and WZ structures, respectively. The 12xx and 13xx bond families represent the short-range order by forming rhombus clusters, usually considered as a sign of a disordered system^23^. Figure 3 represents the RDF and HA indices for the core atoms of 20 Å NPs (*r* < 5). Both RDF and HA were calculated from the trajectories of the last 1 ns of simulations.

**Figure 4:**
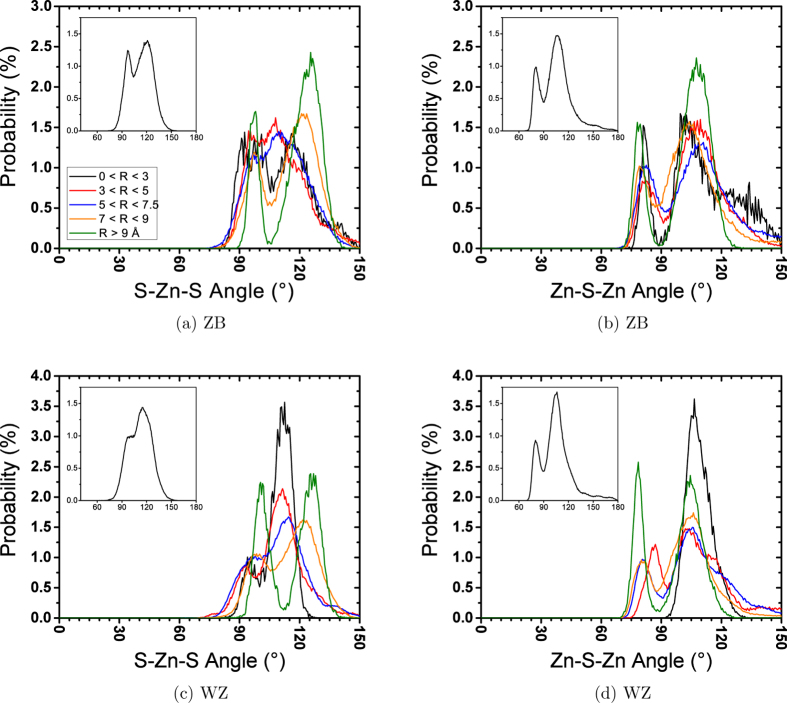
Angular distribution over the last 1 ns of relaxation at 300 K for 20 Å NPs with initial (a,b) ZB and (c,d) WZ structures. Different colours correspond to different distances of the vertex of the angle (central atom) from the centre of the NP. Inset plots are overall angular distributions.

Figure 3 shows that structures of the cores of both NPs considerably diverge from the initial crystal structure. A large number of 1201 and 1311 bond types also indicates a highly distorted structure. Although almost all central atoms are still 4-coordinated in the relaxed 20 Å NPs, they have lost their initial crystal structure. Apparently, the structural change is more severe in the ZB structure. This can be deduced from the stronger first RDF peak and lower number of 1201 and 1311 bonds in WZ NP (Fig. 3b). The tiny peak before the first neighbour peak is also observed in the core Zn-Zn RDFs, showing that 4-rings have also formed in the 4-coordinated cores of 20 Å NPs. The formation of 4-rings has been reported in the crystallization of ZnS from an amorphous structure^24^ and simulated annealing^20^. This peak is missing in the S-S RDFs because 4-rings adopt a rhombus shape in which S atoms stay farther from each other. The bigger length of S-S pairs occurs because repulsive forces are stronger between anions in ionic components. In the ZB and WZ structures, Zn-Zn and S-S distances are identical, but once NP loses its crystal structure, S ions have the opportunity to repel each other and stay further away causing Zn-Zn distances to decrease. This explains why Zn-Zn RDF peaks for 10 and 20 Å NPs shift to the right compared to the unrelaxed RDF peaks (Fig. 1). The deviation from the ideal crystal structure becomes more severe by coming closer to the surface, where the atoms have more freedom to move, i.e., the S-S distances increase more and Zn-Zn distances become shorter. When the atomic arrangement deviates from the nominal crystal structure of the NPs, the atomic bond angles are also altered. Perfect ZB and WZ crystals show a single peak angular distribution with a maximum located at 109.47°, which is the tetrahedral bond angle. Any deviation from this angular distribution is an indication of deviation from the crystal structure. Figure 4 shows the distribution of Zn-S-Zn and S-Zn-S angles at different distances from the centre of 20 Å ZB and WZ NPs.

**Figure 5:**
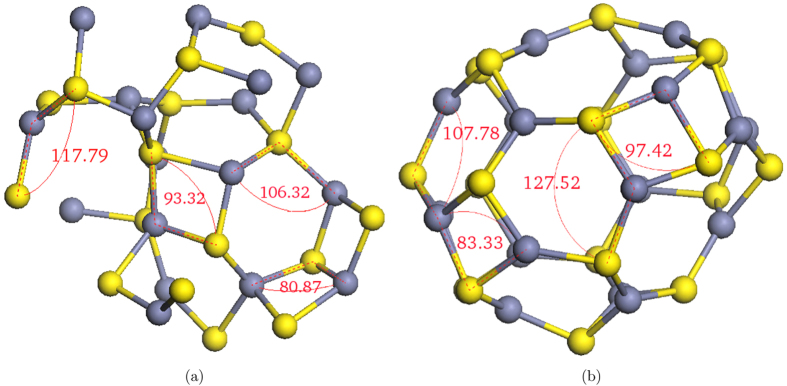
Final configurations of (a) atoms at the core of 20 Å ZB NP (*r* < 5) and (b) 10 Å ZB NP (yellow and grey spheres are S and Zn ions, respectively).

While a small portion of atoms at the centre of the 20 Å WZ NP (*r* < 3) show a tetrahedral angular distribution (Fig. 4c,d), ZB NP shows a completely distorted angular distribution, even for the central atoms. This further confirms that the tetrahedral bond structure is more stable in WZ NPs. Similar to Fig. 3, transformation from the single-peak to the double-peak angular distribution in Fig. 4 shows that the atomic structure of the cores of 20 Å NPs deviates from the tetrahedral structure, although atoms are still 4-coordinated. In the double peak angular distribution, the first peak corresponds to the formation of 4-rings where the angles are smaller. Generally, Zn-S-Zn angles are smaller than S-Zn-S angles because once the deviation from the tetrahedral bond structure occurs, Zn-Zn and S-S distances become shorter and bigger than their equilibrium distances in the crystal structure (the first peak in the unrelaxed RDF), respectively. As mentioned before, this deviation becomes more significant closer to the surface where atoms have more freedom to move. This movement causes the S-Zn-S and Zn-S-Zn angular distributions to move to the right and the left, respectively. The intensity of the 4-rings’ peak in the angular distribution becomes significantly greater when R > 9 Å showing the higher probability of forming 4-rings on the surface. Figure 5 shows the final configurations of 10 Å and the core of 20 Å ZB NPs. All atoms at the core of 20 Å ZB NP are 4-coordinated, while the 10 Å ZB NP final configuration is made entirely of 3-coordinated atoms. Figure 5 shows that the S-Zn-S and Zn-S-Zn atomic angles between the 3-coordinated atoms on the surface differ from the 4-coordinated atoms in the core.

**Figure 6:**
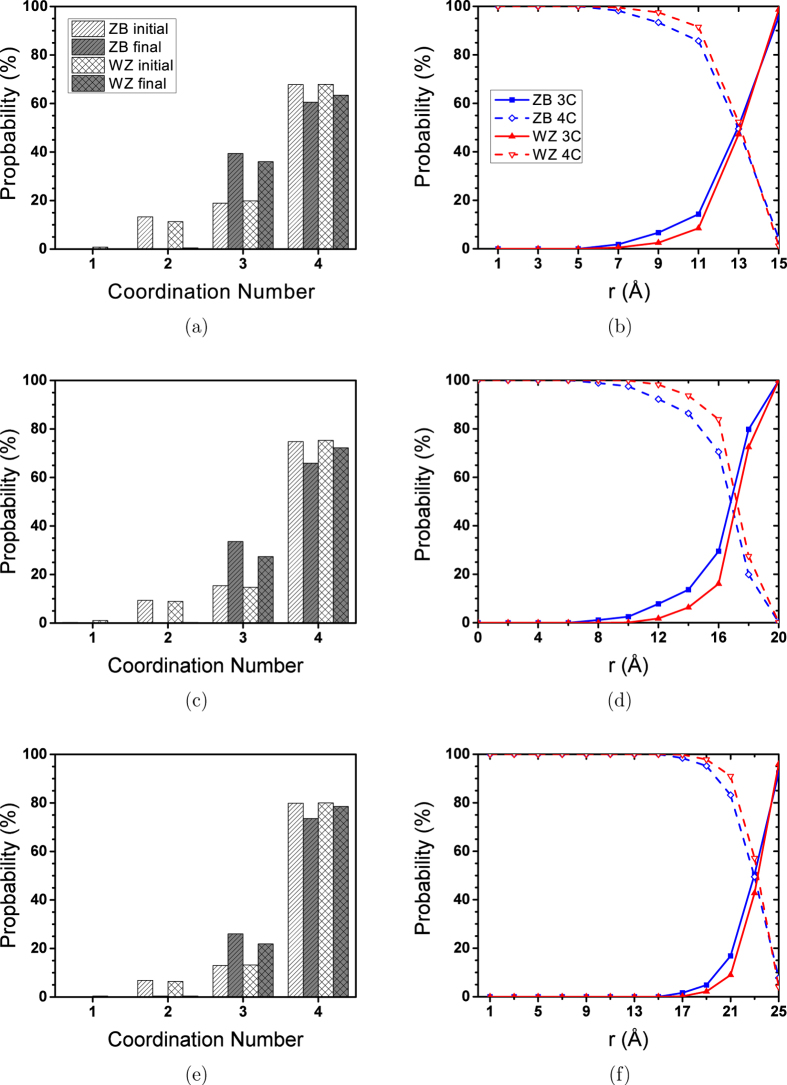
CN distribution for (a,b) 30 Å, (c,d) 40 Å, and (e,f) 50 Å NPs. Bar plots (left column) show the probability of finding atoms with different CNs in the initial and final configurations. Line plots (right column) show probability of finding a 4− or 3-coordinated atom at different distances from the centre during the last 1 ns of simulations.

*3 to 5 nm NPs.*    The above analyses confirm that the structure of the 20 Å ZnS NPs consists of 3-coordinated atoms on the surface and a distorted structure of 4-coordinated atoms in the core. The 4-coordinated atoms mainly lose their tetrahedral structure and arrange into 4- and 6-rings. However, small number of atoms at the core of 20 Å NP (*r* < 3 Å) with the WZ initial structure still keep their tetrahedral arrangement. The initial structure seems to completely fade away in the ZB NP. Based on these observations, we can expect bigger NPs to have a structure, including the 4-coordinated atoms with the tetrahedral arrangement at the core, followed by a shell that includes the distorted structure of 4-rings and 6-rings of 4-coordinated atoms which connects 3-coordinated surface atoms to the core. The same analyses were performed to study the structural changes in the bigger NPs. Figure 6 shows the CN distribution of 30, 40 and 50 Å NPs.

**Figure 7:**
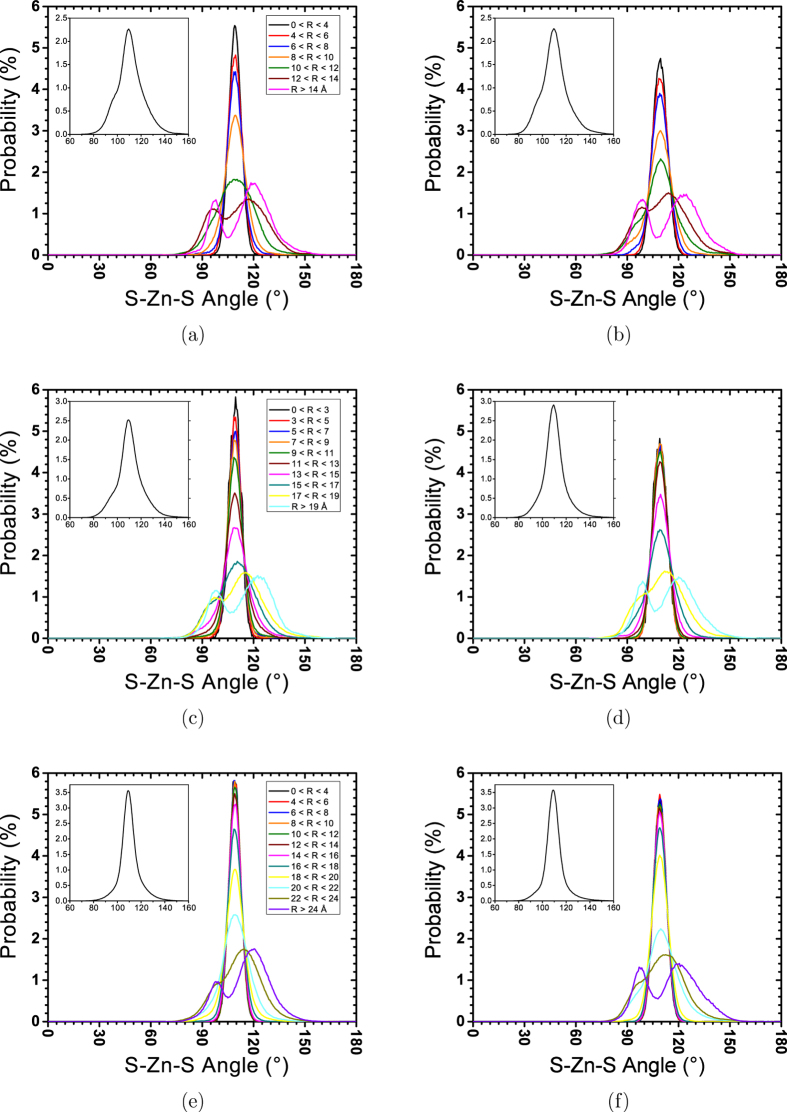
Angular distribution over thr last 1 ns of relaxation at 300 K for 30 Å NPs with initial (a,b) ZB and (c,d) WZ structures. Different colours correspond to different distances of the vertex of the angle (central atom) from the centre of the NP. Inset plots are overall angular distributions.

Comparing Fig. 6 with Fig. 2b, we can see that unlike the 20 Å NPs, the 4-coordinated atoms are dominant in bigger NPs. The stability of the 4-coordinated structure increases by increasing the size of NPs. Similar to what was observed in 20 Å NP, the ZB structure is less stable than the WZ structure. Figure 6b, d, f show that a 50% probability for 3− and 4-coordinated atoms is located at around 1 Å beneath the surface. This distance, which was also observed for 20 Å NPs (Fig. 2c), shows that in all NPs ranging in size from 20 Å to 50 Å, the 3-coordinated structure is only limited to the surface. However, in 20 Å NPs, the surface relaxation causes a pervasive structural change, resulting in the disappearance of the tetrahedral structure. Thus, it seems that using the probability of finding 3-coordinated atoms in NPs is not a good way to quantify the stability of tetrahedral structure. As when we analyzed for 20 Å NPs, we calculated the angular distribution in different distances from the centre of NPs to track the change in tetrahedral structure. Figure 7 shows the S-Zn-S angular distribution calculated using trajectories of the last 1 ns of simulations.

Figure 7 shows that all NPs with sizes ranging from 30 Å to 50 Å have shown strong tetrahedral peaks at their centres. Compared to WZ NPs, the tetrahedral angular distributions of the central atoms in ZB NPs have a higher intensity. However, the intensity of the tetrahedral peak in ZB NPs decreases at a higher rate as it approaches the surface. The lower intensity of the tetrahedral angular distribution at the centre of WZ NPs is probably due to the anisotropic thermal vibration in WZ crystal, which causes the tetrahedral angle to vibrate in a wider range. The anisotropy of thermal vibration of WZ ZnS has been reported in DFT calculations^25^. Moreover, it is well known that real WZ lattices of II-VI semiconductors deviate from the ideal WZ structure^26^. It has been already shown that the potential formulation used in this study successfully reproduces the real WZ structure of ZnS at 300 K^27^. This difference can make the angle of the tetrahedral bond deviate slightly from the ideal tetrahedral angle (109.47 Å) in the real WZ structure. This effect is also the main reason of the different polar behaviour of ZB and WZ structures. This behaviour is explained in more details later. Except for atoms at the very centre of NPs (*r* < 5 Å), all other tetrahedral angular distributions in 30 and 40 Å WZ NPs are stronger than those in ZB NPs. This suggests that WZ NPs have a more stable tetrahedral structure.

As shown in Fig. 6, 3-coordinated atoms become dominant from almost 1 Å underneath the nominal radius of NPs. In Fig. 7, we can see that these atoms show a double-peak angular distribution in which both peaks have almost the same intensity. The first peak is due to the formation of 4-rings on the surface. In all diagrams, we can see that the deviation from single-peak to double-peak distribution starts from almost 5 Å underneath the surface. Since Fig. 6 shows that almost all atoms are still 4 coordinated at this distance, the formation of the double-peak distribution can be attributed to the distorted 4-coordinated structure (similar to what we observed at the core of 20 Å NPs). Double-peak distributions move to the right as we approach to the surface. This is because the S-Zn-S angles become bigger on the surface as atoms have more freedom to move.

**Figure 8:**
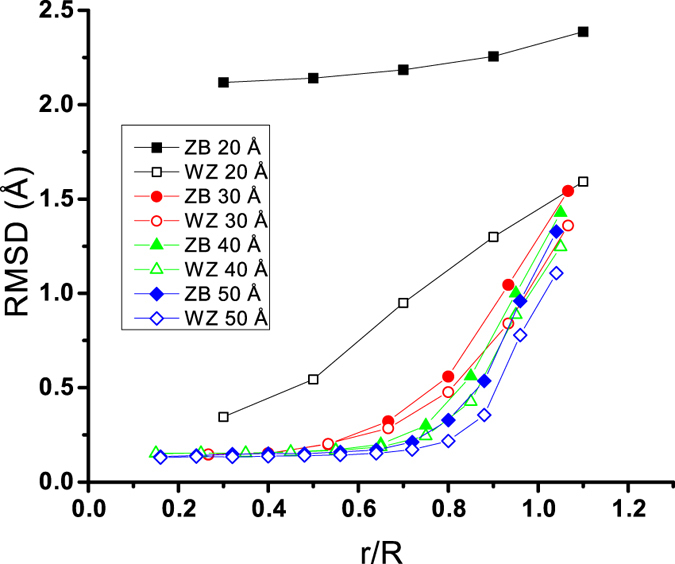
RMSD calculated for atoms located in a shell confined between r and r - 2 Å. R is the radius of the NP.

*Root Mean Square Displacement.*    To verify the formation of the distorted 4-coordinated structure under the surface, we also calculated the root mean square displacement of atoms with respect to their initial positions. In RMSD calculations the position of atoms in each time step is corrected to remove the NP’s centre of mass rotation and movement effect. Figure 8 shows the average RMSD of atoms at different distances from the centre.

Apparently, the RMSD of centres of all NPs is negligible except for 20 Å NPs. This verifies the angular distribution results which showed that all NPs except for the 20 Å NPs kept their tetrahedral structures at their centres. Excluding the 20 Å NPs, Fig. 8 also shows that RMSD plots for all NPs start to diverge from the plateau from about 5 Å beneath the surface. As mentioned before, the large displacement of atoms in this region can be a sign that distorted 4-coordinated structure was formed. The formation of distorted 4-coordinated structure was also characterized by the appearance of double-peak angular distributions in Fig. 7.

**Figure 9:**
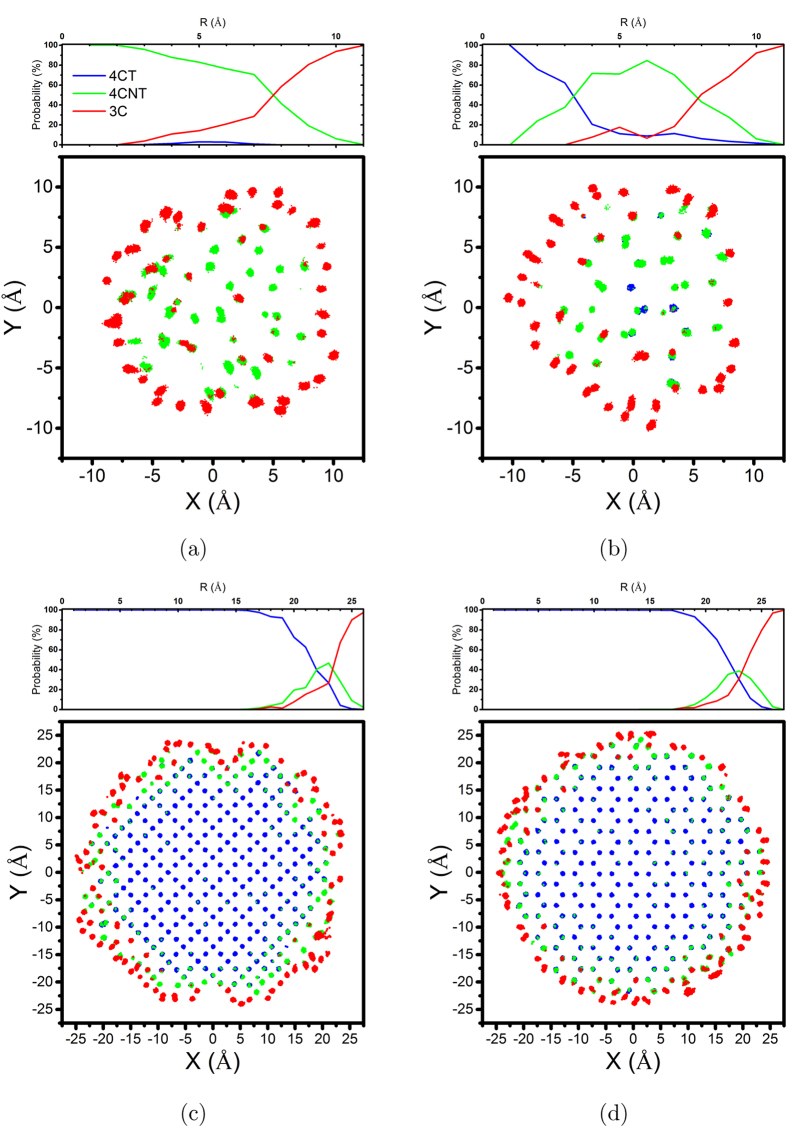
Representation of three atom types in the last 1 ns of relaxation of (a) 20 Å ZB, (b) 20 Å WZ, (c) 50 Å ZB and (d) 50 Å WZ NPs. 4CT: 4-coordinated atoms whose all bond angles are between 100° and 120° (tetrahedral atoms), 4CNT: 4-coordinated atoms which have at least one angle out of the aforementioned range (not tetrahedral 4-coordinated atoms), and 3C: 3-coordinated atoms (surface atoms). Scatter plots show the distribution of different atom types in a slab with −2 < *Z* < 2 and line plots show the probability of finding each atomic type in a shell confined between R and R - 1 Å. Different atomic types are distinguishable by different colours.

*3-Phase Structure.*    The above observations and analyses confirm the three-phase structure of ZnS NPs: a) a crystalline core which maintains the initial tetrahedral bond structure, b) a distorted network of 4-coordinated atoms which surrounds the crystalline core, and c) a surface structure which includes 4 and 6-rings of 3-coordinated atoms. We further quantify these three phases using the bond angle and coordination number of each atoms as follows: 4-coordinated atoms whose all bond angles range between 100° and 120° are considered as tetrahedral atoms (4CT); the 4-coordinated atoms which have at least one angle out of the aforementioned range are categorized as not tetrahedral 4-coordinated atoms (4CNT); and finally, the 3-coordinated atoms (3C) which form the surface structure. According to the previous analyses, all atoms in the relaxed ZnS NPs should belong to one of these categories. Results of these calculations are illustrated in Fig. 9 using two different graphical representations. Line plots show the probability of finding each atom type at different distances from the centre. Each dot in the scatter plots represents an atomic position during the last 1 ns of simulations. For clarity, only the position of atoms located in the central slab (−2 Å < *Z* < 2 Å) of NPs is shown. Moreover, to avoid redundancy, only the results for 20 and 50 Å NPs are represented.

**Figure 10:**
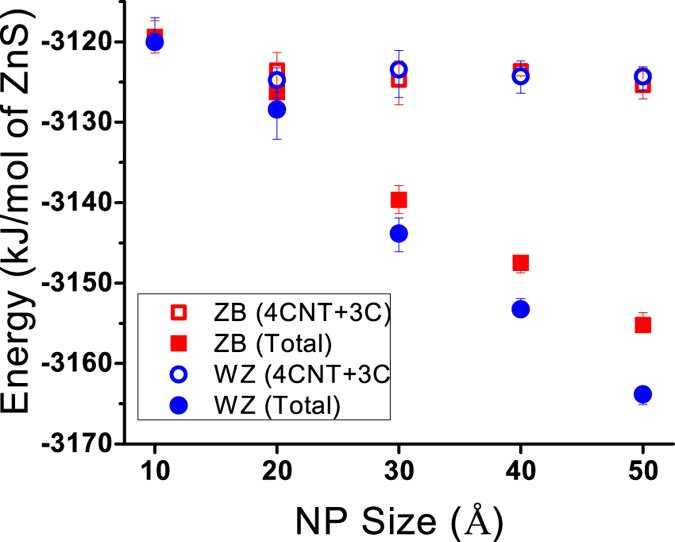
The average potential energy of ZnS NPs after relaxation. The energy of the surface layers (4CNT + 3C ions) was calculated via subtracting the energy of the crystalline core from the total energy of the NP.

Figure 9a confirms the complete disappearance of the tetrahedral structure in the 20 Å ZB NP. In contrast, a small portion of tetrahedral atoms is detected in the centre of 20 Å WZ NP. Figure 9c, d also show that the deviation from the tetrahedral structure begins from about 5 Å beneath the surface. This 5 Å thickness of distorted structure, which has also been observed in Fig. 8, suggests that 30 to 50 Å ZnS NPs, regardless of their size or initial crystal structure, experience a similar surface relaxation which results in a network of 3-coordinated atoms on the surface followed by a couple of layers of distorted 4-coordinated atoms. This similarity is of great importance because it can result in the same surface properties that can control the interaction of NPs with the surrounding environment. Atoms located on the edges and corners of surface facets are more under-coordinated (1− and 2-coordinated) and more prone to chemical and electrochemical reactions^28^. The surface behaviour can be greatly changed if the faceted nature of the NPs’ surfaces disappears, and the coordination number of all the atoms on the surface is turned to three. Figure 10 confirms that the similar surface structures of ZnS NPs leads to similar surface energies as well. The energy of the surface atomic layers including 4CNT and 3C atoms was estimated by subtracting the total energy of NP from the energy of the crystalline core. The energy of the ZB and WZ lattices has been calculated from NPT simulations of the periodic bulk structures. The energy of the crystalline core of NPs was then calculated by multiplying the number of 4CT atoms by the energy per atom obtained from the simulation of the bulk structures. The average number of 4CT, 4CNT and 3C atoms and the average total energy of NPs were calculated from the last 1 ns of simulations.

Figure 10 shows that the total energy of a NP decreases as the size increases. This is expected, as by increasing the size, the surface-to-volume ratio decreases and the structure of the NP also transforms from more amorphous-like to more crystalline-like, and energy approaches the level of bulk energy. However, the energy of the surface atoms (open symbols) is independent of both the initial crystal structure and the size of the NP.

**Dipole Moment.**    The dipole momentum is calculated in this study to examine the effect of structural change. Dipole-dipole interactions are of a great importance as they are shown to be the governing factor in the agglomeration^29^, oriented attachment^30^, and stabilization of superlattice structures^31^ of non-metallic NPs. There has been considerable number of studies on finding the origin of large permanent dipole in ionic NPs. A large DM is expected in WZ NPs due to the polar nature of WZ lattice but the DM is expected to be absent in ZB NPs, due to the *T*_*d*_ symmetry of ZB lattice. Li and Alivisatos’s study on CdSe nanorods showed that a permanent DM was proportional to the volume of nanorods. They considered the origin of the DM was due to the natural polar character of the WZ structure^32^. Nann and Schneider have also shown that small crystallographic deviations from the ideal WZ structure could result in a large permanent dipole moment^26^. In contrast, Shim and Guyot-Sionnest have shown a large DM in both WZ CdSe and ZB ZnSe nanocrystals which linearly depended on the radius^33^. Since the large DM has been observed for both ZB and WZ structures, they concluded that this linear size dependence was not due to the polar character of the WZ lattice but to the faceted surface structure of nanocrystals. A subsequent study of Shanbhag and Kotov showed that minor deviations from a symmetric tetrahedral shape of ZB CdS nanocrystals could result in large DMs^34^. Cho *et al.* have shown that the dipole moment of PbSe nanocrystals with a centrosymmetric rocksalt lattice was large enough to result in the formation of nanowires through the oriented attachment of nanocrystals^35^. Considering a random distribution of polar facets and the probability of lacking of central symmetry, they showed that about 89% of possible shapes of PbSe nanocrystals was polar. The self-assembly of ZnS nanocrystals into ellipsoidal shapes has also been explained by charge-charge, charge-dipole, and dipole-dipole interactions of non-symmetric ZnS NCs along the [111] direction^36^.

**Figure 11:**
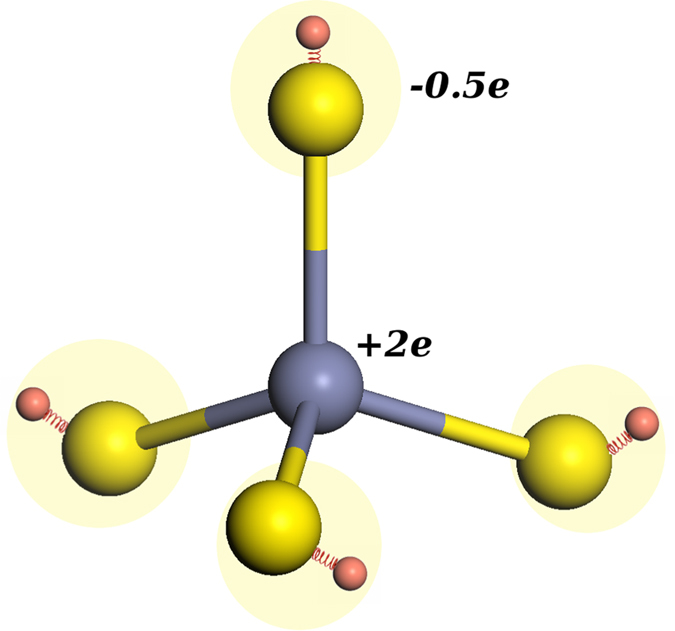
Tetrahedron model used to calculate the bulk dipole moment.

All the previous studies explain the dipole moment in NCs with a cubic crystal lattice via the deviation of distribution of surface ions from the central symmetry. In contrast, WZ NCs polarity is mainly related to deviation of WZ lattice from the idea tetrahedral bond structure. In this study we will systemically quantify the effect of surface and bulk asymmetry of ZnS NPs on the total DM. We also propose the dynamical structural relaxation of NPs (which is explained in previous section) as another major parameter controlling the DM. We used MD trajectories to calculate the total DM of a NP as


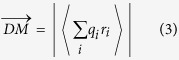


where *q*_*i*_ and *r*_*i*_ are the charge and the position vector of ion i, respectively. To study the effect of the crystal structure of NPs on their final DM, the natural DM of ZB and WZ lattices was calculated first using trajectories of the 1 ns NPT simulation of bulk structures at 300 K and 0 atm. We used the simple-point charged model of Nann and Schneider^26^ to calculate the bulk DM. In this model, the DM is calculated for each Zn-S tetrahedron unit as shown in Fig.11. In each Zn-S tetrahedron, the Zn ion caries the elemental charge of +2e and each S ion (core plus shell) caries 25% of the Zn charge. For an ideal tetrahedral bond structure, where all atomic bond lengths are equal and all bond angles are equal to 109.47°, the DM is equal to zero.

As expected, DM calculations show that bulk ZB structure has no natural DM while the WZ lattice is considerably polar. The average natural DM for each WZ ZnS tetrahedron unit was calculated to be 0.3855 D in the opposite direction of the c axis. This is in an agreement with Nann and Schneider calculations, which confirms the natural DM of real WZ structure as a consequence of the slight *C*_3*v*_-distortion of the elementary tetrahedron in real wurtzite^26^.

**Figure 12:**
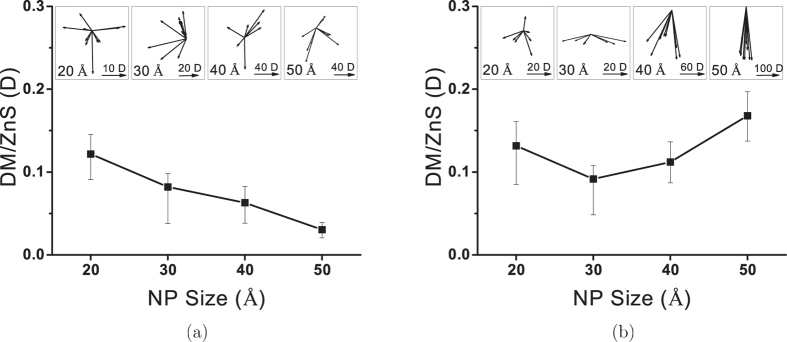
The DM of ZnS NPs calculated using the last 1 ns simulations. (**a**) and (**b**) show the DM of ZB and WZ NPs, respectively. Insets show the projected image of the DM vectors on the XZ plane.

As explained in the Methods section, making initial configurations of NPs is started by cutting spheres from perfect ZB and WZ lattices and followed by removing excessive ions from the surface to achieve charge neutrality. In this paper, when we refer to the initial configurations, we are talking about the initial configurations after charge neutralization. Configurations before the charge neutralization are called as-cut configurations. The charge neutralization of ionic NPs as-cut structures has usually been achieved by randomly removing excessive ions from the surface. However, this method could not ensure the symmetry of the initial configurations. The asymmetry of distribution of positive and negative ions on the surface can cause a DM for initial configurations. We noticed that except for 10 and 20 Å NPs (in which the initial crystal structure is entirely removed after relaxation), the final structures’ DM magnitude and direction is strongly dependent on the initial DM. To study the effect of structural evolution during the NPs’ relaxation on the final DM, we needed to remove this artificial effect of initial structures. We did this using a method other than the random removing of excessive ions from the surface, which results in non-polar initial configurations. Details of our charge neutralization method are explained in the Methods section. For each NP size, 10 different, non-polar initial structures were randomly chosen and relaxed through NVT simulations. Figure 12 shows the DM per ZnS calculated for ZB and WZ NPs using 10 ns NVT simulations at 300 K. Trajectories of the last 1 ns of simulations were used to calculate the DM.

Figure 12 shows polarities of ZnS NPs as a function of NPs size. This figure shows that the DM is evolving differently in ZB and WZ NPs. While the magnitude of the DM per ZnS is decreasing continuously by increasing the size of ZB NPs, it drops and then increases as the WZ NPs size increases. Another major difference between ZB and WZ NPs polar behaviours is the direction of the DM vectors. The directions of DM vectors of relaxed ZB NPs with different initial configurations show no correlation and change randomly. However, the WZ NPs’ DM vectors become more aligned with -Z direction as the size increases. Since the initial structures of NPs are not polar (or negligibly polar in WZ NPs), DMs of final configurations are pure products of structural evolutions of NPs at 300 K. As the ZB lattice remains non-polar at 300 K, divergence from the ideal tetrahedral structure which happens on the atomic layers near surface (aforementioned 4CNT and 3C structures) is the main reason of polarity of relaxed configurations of ZB NPs. The random direction of final DM vectors can also be justified by the fact that the deformed surface structure of a ZB NP has a random atomic arrangement.

For 20 Å WZ NP whose entire tetrahedral structure is almost vanished, the direction of DM randomly changes for different initial configurations similar to ZB NPs. It also seems that the DM magnitude of 20 and 30 Å WZ NPs is similar to the corresponding ZB NPs, but starts to increase afterwards. Unlike the ZB structure, we showed that the WZ lattice is considerably polar at 300 K and the direction of the WZ bulk DM vector is aliened with −Z direction. As the WZ NP becomes bigger, the size of the bulk tetrahedral structure increases (number of 4CT ions) and this will consequently increase the magnitude of the bulk DM in Z direction. This is the reason why DM vectors become more aligned with the Z direction as the size of the WZ NP increases. It also explains why the polar behaviour of 20 and 30 Å WZ NPs, in which deformed surface structure is dominant, is similar to ZB NPs but starts to diverge after 30 Å when the polar crystalline core of WZ NPs becomes the dominant portion of the structure. As the size of the NP increases, the polarity converges to the bulk value. As a result, one should expect that the DM per ZnS magnitude of ZB and WZ NPs approach the bulk values which are 0.0 and 0.3855 D, respectively.

**Figure 13:**
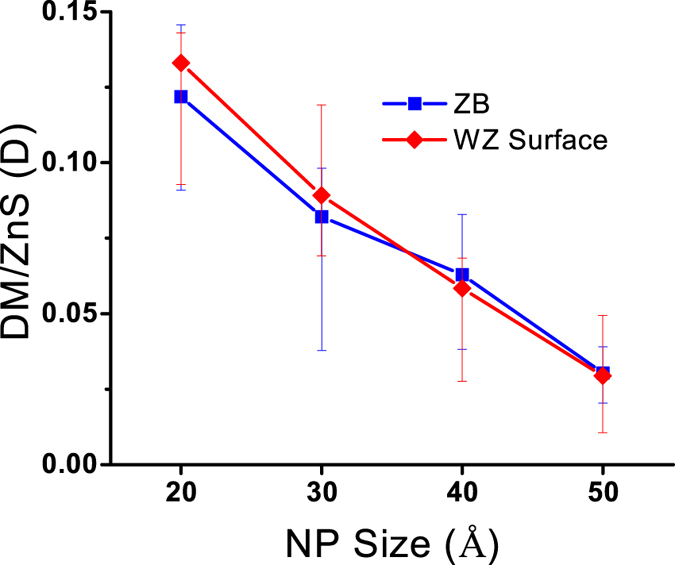
The DM per ZnS caused by the deformed surface structure.

The DM caused by surface atoms of a WZ NP can be calculated by subtracting the DM caused by the crystalline core from the total DM of the NP. The bulk DM of WZ NPs was estimated by multiplying the number of tetrahedral units (number of Zn 4CT atoms) by 0.3855 D (the DM for each tetrahedral unit in the WZ lattice). Figure 13 shows the DM per ZnS caused by the deformed surface structures of WZ NPs along with the total DM of ZB NPs.

Apparently, the magnitude of DM caused by surface structure of WZ NPs is similar to the DM of ZB NPs. This is another indication confirming that the surface structure of ZB and WZ NPs is similar. Figure 13 also shows that the effect of the surface dipole becomes less significant as the size of NPs increases. This is because the deformed surface structure of NPs becomes smaller comparing to bulk tetrahedral structure, as the size of a NP increases.

## Discussion

In this paper, we studied the structural evolution of freestanding ZnS NPs having initial ZB and WZ crystal structures and ranged in size from 1 to 5 nm. We found that except for the 10 and 20 Å NPs, the final configurations of ZnS NPs with both initial crystal structures consisted of three regions: a) a crystalline core which kept the initial tetrahedral bond structure, b) a region of distorted 4-coordinated atoms which formed 4− and 6-rings, and c) 3-coordinated atoms which covered the surface of NPs. In the relaxed structure of 10 Å NPs all of the atoms were 3-coordinated, confirming the formation of the bubble-like structure. For 20 Å NPs, the structure relaxation removed the initial tetrahedral bond structure (region a) entirely. Our structural analyses showed that the surface structure of both ZB and WZ NPs ranging from 2 to 5 nm in size was similar and consisted of 3C and 4CNT atoms. This similarity may result in similar surface properties such as the the surface energy. These analyses also showed that tetrahedral bond structure was more stable in WZ NPs and that ZB NPs experienced more severe structural relaxations.

The effect of the structural evolution of NPs on the dipole moment was also studied. To study the pure effect of structural evolutions, non-polar initial configurations were used for simulations. Results of dipole moment calculations showed that non-polar ideal structures of NPs changed to polar structures after relaxation at 300 K. Since the tetrahedral core of ZB NPs has a non-polar nature, the polarity occurred in these NPs because their surface configurations deviated from the tetrahedral bond structure. There was no specific direction for a dipole moment vector of a ZB NP and the direction changed randomly for different configurations. In contrast, dipole moment vectors of WZ NPs became more aligned with −Z direction as the NP size increased. This was due to the polar nature of WZ lattice, which was shown to have dipole moment of 0.3855 D per ZnS molecule. By increasing the size of the WZ NP, the crystalline polar core became more dominant and controlled the dipole moment magnitude and direction. For NPs smaller than 3 nm, where 4CNT and 3C atoms were predominant, DMs of ZB and WZ NPs were similar due to the similarity of the surface structures. As the size of the NP increased, the 4CT atoms started to control the DM. As a result, ZB and WZ NPs became less and more polar, respectively. By subtracting the dipole moment caused by the crystalline core from the total dipole moment, we calculated the surface dipole moment for WZ NPs. It was shown that surface dipole moment of WZ NPs was in the same range as dipole moment of ZB NPs, which again confirmed the similar surface properties of ZnS NPs in the range of 2 to 5 nm.

## Methods

### Initial configurations construction

The initial configurations of NPs ranging in size from 1 to 5 nm were generated by cutting spheres from perfect ZB and WZ super-lattices. Due to the tetrahedral symmetry of the ZB lattice, for each ion I(x, y, z), there exists a similar symmetric-ion I’(−x, −y, −z) in ZB as-cut configurations. Ions I and I’ make a symmetric-group II’, which has zero DM in all directions (X, Y and Z). Since all ions should belong to a symmetric-group, the total DM of the as-cut configuration would then be zero. WZ belongs to the *C*_3*v*_ point group, which has lower symmetry than *T*_*d*_. While *T*_*d*_ makes DM to be zero in all X, Y and Z directions, *C*_3*v*_ symmetry only ensures zero DM in the X and Y directions. For each ion I(x, y, z) in the *C*_3*v*_ point group, there exist two similar symmetric-ions I’(x’, y’, z) and I”(x”, y”, z) such OI, OI’ and OI” vectors make 120 angles. As the DM of symmetric-group II’I” in the (0001) plane (XY plane) is zero and each ion should belong to a symmetric group, the total DM of WZ the as-cut configuration will be zero in the X and Y directions.

Since the initial configurations of NPs are not necessarily charge-neutral, excess ions were removed from the surface to achieve electroneutrality. The charge neutralization of ionic NPs’ as-cut structures has usually been achieved by randomly removing excessive ions from the surface. However, this method cannot ensure the symmetry of distribution of positive and negative ions on the surface and may result in a DM for initial configurations. In this study, instead of individual ions, symmetric groups were removed randomly from the surface to obtain non-polar initial configurations. It means that excessive ions should be removed in groups of 2 or 3 from ZB and WZ as-cut configurations, respectively. Depending on the total number of excessive ions, complete charge neutrality may not be accessible by removing symmetric ions from the surface. If the total number of excessive ions is not a multiple of two, we will end up having one remaining excessive ion in the NP after removing symmetric groups. Similarly, one or two excessive ions may remain if the total number of excessive ions is not a multiple of three in WZ NPs. In those cases, one or two remaining ions would be removed from the bulk of the as-cut configuration in a way that does not affect the symmetry.

For each of ZB and WZ NPs, 10 initial configurations with the lowest enegies were chosen. As the initial DMs of WZ NPs are not necessarily zero in the Z direction, one more condition was added to enssure that the initial configurations have the lowest DMs (negligible in respect to the final DM after the relaxation). A large number of repetitions (no less than 1,000,000) were performed to make sure that all possible combinations of removing excessive ions from the surface were covered. It is worth noting that initial configurations of WZ NPs were made out of the ideal WZ super-lattice so the probable DM of initial WZ configurations was just due to the asymmetry of distribution of surface ions in the Z direction, but not the asymmetry of the bulk structure. We noticed that for NPs greater than 2 nm, using either the random removing of ions or symmetric groups would not significantly affect the final structure and energy of NPs, but would affect their final DMs.

### Simulation Details

A detailed comparison of the different available ZnS empirical potentials in the literature was published in our previous study^27^. Among all available potentials for ZnS, we chose the potential developed by Hamad *et al.*^37^ for this work. Surface properties are of great importance when studying nanostructures. It has been shown that surface properties calculated by this potential have the best agreement with results of experimental and first principle studies. Furthermore, this potential has successfully reproduced some other ZnS properties such as crystal structures, mechanical properties, thermal expansion and pressure-induced phase transformation. Moreover, developers of the empirical force field used in this study have validated its accuracy in predicting the energy of small ZnS clusters. They did so by comparing the force field results with the first principle calculations^20^,^38^. In their later study, they also emphasized the ability of this force field in modeling the surface properties of the small ZnS clusters by comparing (ZnS)_*n*_ clusters (n = 2–7) optimized with both DFT and IP, which gave a deviation of less than 0.1 Å for bond distances, and less than 51 for angles^17^. Considering the importance of the surface effect in small NPs, we also performed first principle calculations to validate the accuracy of this force field in predicting the energy and structure of 1 nm ZnS NPs. We performed DFT geometry optimization using the DMol^3^ 39,40 code and PW91 exchange-correlation functional^41^ with effective core potentials and a double numerical plus polarization basis set. The energy, structure and dipole moment of 1 nm ZnS NPs were calculated and compared with the empirical interatomic potential results. This comparison, details of which are provided in Supplementary Table S1, shows that the properties of geometry optimized NPs achieved by interatomic potential agree strongly with the DFT results. MD simulations of ZnS NPs were run in vacuum and the canonical ensemble using DL_Poly^42^ simulation code. All simulations were run at 300 K, and the Nosé-Hoover thermostat was used to control the temperature. A time step of 0.5 fs was used, and simulations were carried out for no less than 10 ns.

## Additional Information

**How to cite this article**: Khalkhali, M. *et al.* A size-dependent structural evolution of ZnS nanoparticles. *Sci. Rep.* **5**, 14267; doi: 10.1038/srep14267 (2015).

## Supplementary Material

Supplementary Information
