# Supplementary material for: A size-dependent structural evolution of ZnS nanoparticles
Source: Sci Rep. 2015 Sep 18;5:14267. doi: 10.1038/srep14267 (PMC4585645; doi:10.1038/srep14267)
Supplement: Supplementary Information [file srep14267-s1.pdf]

A size-dependent structural evolution of ZnS nanoparticles

Mohammad Khalkhali[1] , Qingxia Liu[1] , Hongbo Zeng[1] , and Hao Zhang*

[1] Department of Chemical and Materials Engineering, University of Alberta, Edmonton, Alberta T6G 2V4, Canada

*Corresponding author:

Hao Zhang, PhD
Associate Professor

Department of Chemical and Materials Engineering

W7-030 ECERF
Phone: (780) 492-8340
Fax: (780) 492-2881
Email: hao.zhang@ualberta.ca

Table S1: Comparison between DFT and IP (interatomic empirical potential) results for the geometry optimization of the 1 nm zinc blende (ZB) and wurtzite (WZ) ZnS nanoparticles.

Property		ZB	WZ
ΔE_{IP}* (eV)	IP	-1.415	-1.700
	DFT	-1.089	-1.272
ΔE_{DFT} (eV)	IP	-0.859	-0.911
	DFT	-1.03	-1.17
RMSD (Å)	IP	1.63	1.72
	DFT	1.34	1.41
Average Bond Length (Å)	IP	2.321	2.333
	DFT	2.355	2.350
Average Bond Angle (°)	IP	107.913	106.588
	DFT	103.130	103.970
Average Coordination Number+	IP	3.101	3.000
	DFT	2.942	2.955
Dipole Moment++ (D)	IP	1.475	1.9380
	DFT	1.389	1.867

*$\Delta E = E_f - E_0$. Geometry optimization performed using IP and DFT methods. Single point energy calculation was performed for initial and final structures after geometry optimization. Subscript of ΔE shows the method used for the single point energy calculations. In the IP method which includes shell model, we let the shells of S atoms to relax during energy calculations.

+Average coordination numbers of the initial configurations of the ZB and WZ nanoparticles were 2.895 and 2.636, respectively.

++Dipole moment was calculated using the DFT method.
